# Isolation and Characterization of *SPOTTED LEAF42* Encoding a Porphobilinogen Deaminase in Rice

**DOI:** 10.3390/plants12020403

**Published:** 2023-01-15

**Authors:** Lin Liu, Yunpeng Wang, Yunlu Tian, Shuang Song, Zewan Wu, Xin Ding, Hai Zheng, Yunshuai Huang, Shijia Liu, Xiaoou Dong, Jianmin Wan, Linglong Liu

**Affiliations:** 1 State Key Laboratory for Crop Genetics and Germplasm Enhancement, Jiangsu Plant Gene Engineering Research Center, Nanjing Agricultural University, Nanjing 210095, China; 2 National Key Facility for Crop Gene Resources and Genetic Improvement, Institute of Crop Science, Chinese Academy of Agricultural Sciences, Beijing 100081, China

**Keywords:** chlorophyll biosynthesis, chloroplast, rice (*Oryza sativa* L.), RNA editing, spotted leaf, porphobilinogen deaminase (PBGD)

## Abstract

The formation and development of chloroplasts play a vital role in the breeding of high-yield rice (*Oryza sativa* L.). Porphobilinogen deaminases (PBGDs) act in the early stage of chlorophyll and heme biosynthesis. However, the role of PBGDs in chloroplast development and chlorophyll production remains elusive in rice. Here, we identified the *spotted leaf 42* (*spl42*) mutant, which exhibited a reddish-brown spotted leaf phenotype. The mutant showed a significantly lower chlorophyll content, abnormal thylakoid morphology, and elevated activities of reactive oxygen species (ROS)-scavenging enzymes. Consistently, multiple genes related to chloroplast development and chlorophyll biosynthesis were significantly down-regulated, whereas many genes involved in leaf senescence, ROS production, and defense responses were upregulated in the *spl42* mutant. Map-based cloning revealed that *SPL42* encodes a PBGD. A C-to-T base substitution occurred in *spl42*, resulting in an amino acid change and significantly reduced PBGD enzyme activity. SPL42 targets to the chloroplast and interacts with the multiple organelle RNA editing factors (MORFs) OsMORF8-1 and OsMORF8-2 to affect RNA editing. The identification and characterization of *spl42* helps in elucidating the molecular mechanisms associated with chlorophyll synthesis and RNA editing in rice.

## 1. Introduction

The chloroplast is an important organelle for photosynthesis in higher plants. Its main function is to convert light energy into chemical energy through chlorophyll. Chlorophyll is involved in the absorption, transmission, and conversion of light energy. It is a long-term breeding objective to improve the chloroplast and chlorophyll photosynthesis capability.

So far, many genes related to chlorophyll synthesis in rice (*Oryza sativa*) have been cloned, including glutamyl-tRNA synthetase (OsGluRS), magnesium ion chelatase (Chl), protochlorophyll ester oxidoreductase (OsPORB), porphobilinogen deaminase (PBGD), and so on. OsGluRS is the first enzyme in the chlorophyll synthesis pathway in plants. When OsGluRS is mutated, plant leaves turn yellowish green while plant growth and development are inhibited. [1]. Chl contains three subunits, ChlH, ChlD, and ChlI [2,3]. Mutations in the former two gene results in a yellow-green phenotype, whereas mutating the last one (ChlH) affects the chloroplast structure and chlorophyll biosynthesis, resulting in plant death four weeks after germination [4,5]. Likewise, the abnormal expression of OsPORB produces yellow, white, and necrotic spots on the leaves [6]. PBGD is an enzyme that functions during the early stage of chlorophyll and heme biosynthesis. PBGD catalyzes the deamination of four porphobilinogen molecules and their gradual polymerization into linear hydroxymethylbilane. A loss-of-function mutant of PBGD in maize (cf1) exhibits yellowing and even necrotic leaves. The necrotic spotted leaf phenotype of the cf1 mutant is caused by the decreased activity of tetrapyrrole synthetase, accompanied by reactive oxygen species (ROS) burst and the disturbance of the ROS scavenge system [7]. Another loss-of-function mutant of PBGD in Arabidopsis, rugosa1, showed spontaneous lesions reminiscent of those seen in lesion-mimic mutants (LMM), such as irregularly shaped leaves and a reduced growth. [8,9].

An LMM often exhibits hypersensitive responses in the absence of pathogens, resulting in the production of disease-resistant substances such as ROS, plant hormones, and phenolic complexes [10,11]. By screening rice brown necrotic spot mutants, Wang et al. identified an LMM-related gene, *OsCNGC9*. This gene relies on calcium ion channels to stimulate the plant’s own immune system, thereby regulating innate immunity in rice [10]. Another rice LMM, *apoptosis leaf and sheath 1* (*als1*), showed the accumulation of jasmonic acid (JA) and salicylic acid (SA) and an increased resistance to rice fungus, indicating that *ALS1* is involved in the defense responses mediated by SA and JA [11]. Moreover, most of the rice LMMs, such as *spl2*, *spl7*, *spl11*, *dj-lm*, and *pls3*, accumulate ROS (such as H_2_O_2_ and O^2−^) in the lesion sites, thereby negatively affecting the chloroplast development [12,13,14,15,16,17]. Despite the clarification of the various PBGDs in the chloroplast development of maize and Arabidopsis, the role of PBGD in rice defense responses and chloroplast development remains elusive.

As a common processing event of organellar transcripts in higher plants, RNA editing is essential for gene repair and plant development [18,19,20,21]. The mutation of the RNA recognition motif (RRM) protein ORRM1, which was involved in the RNA editing of plant organelles, leads to the complete loss of the functions of 12 editing sites in Arabidopsis [22]. ORRM1 interacts with multiple organelle RNA editing factors (MORFs), MORF2 and MORF8/RIP1, to regulate the development of chloroplasts and mitochondria [22]. The RanBP2 zinc finger protein family member OZ1 affects the expression of the MORF protein by interacting with ORRM1 [23]. Although MORF and RRM play crucial roles in RNA editing in mitochondria and plastids [24,25], other factors such as PBGD have not been studied in detail.

In this study, we identified the *spotted leaf 42* mutant (*spl42*) in rice. The *spl42* mutant displayed an irregular reddish-brown blotchy phenotype at the third to fourth leaf stage, which was accompanied throughout the whole growth period. Map-based cloning revealed that *SPL42* locates on the short arm of chromosome 2 and encodes a PBGD family protein. Transgenic experiments confirmed that the functional defect of PBGD was the cause of the *spl42* mutant phenotype. The aim of this study is to reveal the role of PBGD in chloroplast development and RNA editing in rice. At the same time, the characterization of the lesion-mimic mutant *spl42* helps to decipher the molecular mechanism of the defense response. This study helps in stabilizing the production of rice, a major staple food crop for more than half of the world’s population.

## 2. Results

### 2.1. Phenotypic Characteristics of the spl42 Mutant

**Figure 1:**
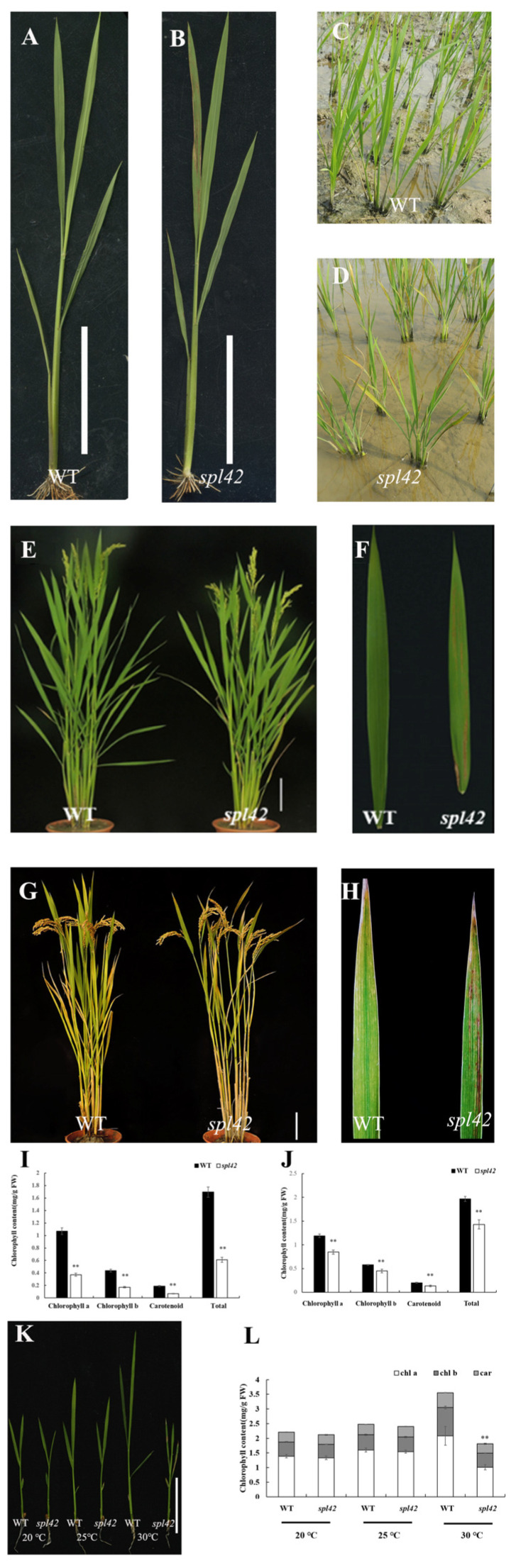
Phenotypic characterization of the *spl42* mutant. (**A**,**B**) Individual images of wild type (WT, left) and *spl42* (right) at the four-leaf stage. Scale Bar = 5 cm. (**C**,**D**) Field phenotypes of wild type (left) and *spl42* (right) two weeks after transplanting into the field. (**E**,**F**) Images of individual plants (left) and flag leaves (right) of wild type and *spl42* at heading stage. Scale Bar = 10 cm. (**G**,**H**) Images of individual plants (left) and flag leaves (right) of wild type and *spl42* at the mature stage. Scale Bar = 10 cm. (**I**,**J**) The chlorophyll contents of wild type and *spl42* at the seedling (left) and heading stages (right). Error bars represent ± SD (*n* = 3). (**K**) Seedling phenotypes of wild type and *spl42* under different temperature treatments. Scale Bar = 5 cm. (**L**) The chlorophyll content of wild type and *spl42* under different temperature treatments. Chl a: Chlorophyll a; Chl b: Chlorophyll b; Car: total carotenoids. Error bars represent ± SD (*n* = 3). Asterisks indicate a significant difference between the wild type and *spl42* plants by Student’s *t*-test, ** *p* < 0.01.

The *spl42* mutant was isolated from the ethyl methane sulfonate (EMS)-mutagenized japonica variety Ningjing6 and designated according to the leaf phenotype. Under field conditions, *spl42* exhibited an irregular reddish-brown spotted leaf phenotype at the third or fourth leaf stage, which then spread to the whole leaf (Figure 1A,B). Two weeks after transplanting in the field, the old leaves of the *spl42* mutant were full of lesion spots, but the new leaves showed fewer spots (Figure 1C,D). As the plant developed, the spots on the blade of the *spl42* mutant continued to spread, and the mature leaves of *spl42* demonstrated an early senescence symptom during anthesis (Figure 1E–H). To further characterize the *spl42* mutant, the chlorophyll and carotenoid contents were measured at the seedling and heading stages. The contents of chlorophyll a and b and carotenoids in the *spl42* mutant were significantly lower than those of the wild type at both seedling and heading stages (Figure 1I,J). The mature *spl42* mutant plants displayed a significant reduction in the plant height, panicle length, flag leaf length, and seed setting rate compared with the wild type (Table A1).

Since temperature contributes to the formation of lesion-like symptoms [13], we investigated the phenotype of the *spl42* mutant under three temperature gradients (20 °C, 25 °C, and 30 °C; see the Materials and Methods section for the treatment conditions) and measured the chlorophyll contents. The *spl42* mutant under high-temperature conditions (30 °C) exhibited more severe red-brown spots than the wild type, accompanied by growth inhibition. In agreement with the lesion phenotype, chlorophyll a, chlorophyll b, and the carotenoids in the mutant were decreased significantly (Figure 1K,L). On the contrary, the *spl42* exhibited a leaf color similar to that of the wild type at low temperatures (20 °C and 25 °C) with no difference in the chlorophyll content (Figure 1K,L). These results suggested that *spl42* was sensitive to high-temperature stresses.

### 2.2. Chloroplast Structures and Related Gene Expression of the spl42 Mutant

**Figure 2:**
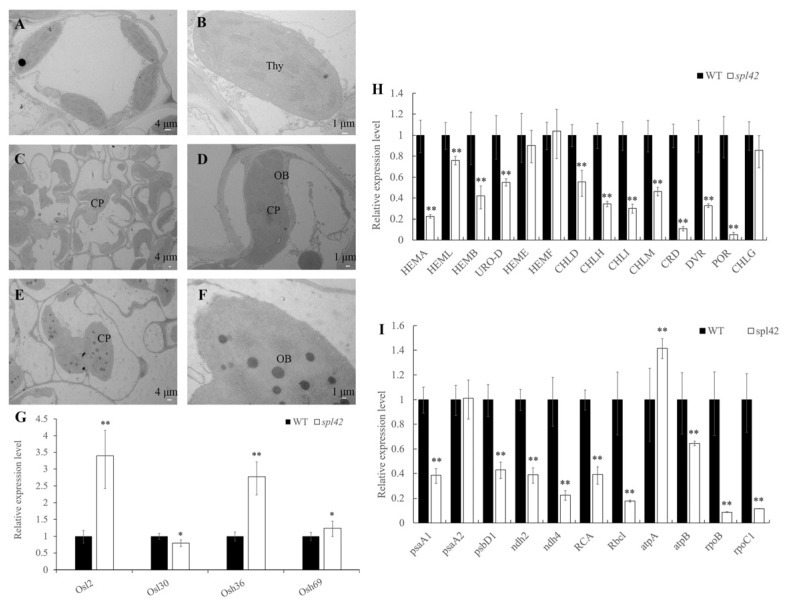
Transmission electron microscopic observation of the wild type (WT) and the *spl42* mutant and related gene expression analysis. (**A**,**B**) Chloroplast ultrastructure of wild-type leaves at four-leaf stage. Scale Bar = 4 μm in (**A**) and 1 μm in (**B**). (**C**,**D**) Ultrastructure of the chloroplast in green part of leaves of *spl42* at four-leaf stage. Scale Bar = 4 μm in (**C**) and 1 μm in (**D**). (**E**,**F**) Ultrastructure of the chloroplast in the spotted part of *spl42* at the four-leaf stage. Thy: thylakoid lamellar; CP: chloroplast; OB: osmophilic. Scale Bar = 4 μm in (**E**) and 1 μm in (**F**). (**G**) Relative expression analysis of the genes related to leaf senescence in the wild type and the *spl42* mutant. Error bars represent ± SD (*n* = 3). (**H**) Relative expression analysis of the genes related to chloroplast development in the wild type and the *spl42* mutant. Error bars represent ± SD (*n* = 3). (**I**) Relative expression analysis of the genes related to chlorophyll biosynthesis in the wild type and the *spl42* mutant. Error bars represent ± SD (*n* = 3). Asterisks indicate a significant difference between WT and the *spl42* plants by Student’s *t*-test, ** *p* < 0.01; * *p* < 0.05.

Considering that the *spl42* spotted leaf phenotype might be related to the abnormal development of chloroplasts, we observed the chloroplast ultrastructure of four-leaf seedlings by transmission electron microscopy (TEM). In wild-type seedlings, the chloroplasts developed normally and the thylakoid structures were intact and closely arranged (Figure 2A,B). However, two types of chloroplasts were observed in mutant leaves: the first type was from the green area of the spotted leaf where the chloroplasts were larger than those of the wild type, and the thylakoids were relatively loosely arranged (Figure 2C,D). The second type was the spotted leaf part of the *spl42* mutant, where the chloroplasts were broken and degraded (Figure 2E,F).

Based on the spotted leaf phenotype of the *spl42* mutant, we used qRT-PCR to analyze the expression of genes related to leaf senescence in wild-type and *spl42* mutant plants at the four-leaf seedling stage. Except for *Osl30*, the expression levels of other leaf senescence genes were significantly up-regulated (Figure 2G). We also analyzed the expression of genes related to the chloroplast development. Most of the genes involved in the chloroplast development were significantly down-regulated, such as *psaA1* (encoding photosystem I subunit A1), *psbD1* (encoding photosystem I subunit D1), *ndh2* (encoding NADH dehydrogenase subunit 2), *ndh4* (encoding NADH dehydrogenase subunit 4), *RCA* (encoding rubisco activase), *RbcL* (encoding rubisco large subunit), *atpB* (encoding ATP synthase subunit B), *rpoB* (encoding RNA polymerase subunit B), and *rpoC1* (encoding RNA polymerase subunit C1) (Figure 2H). These results were consistent with the inhibition of chloroplast development in the *spl42* mutant. In addition, the reduced expression levels of the genes involved in the chlorophyll synthesis indicated that the chlorophyll synthesis was severely blocked in the *spl42* mutant (Figure 2I).

### 2.3. ROS Accumulation Is Elevated in the spl42 Mutant

**Figure 3:**
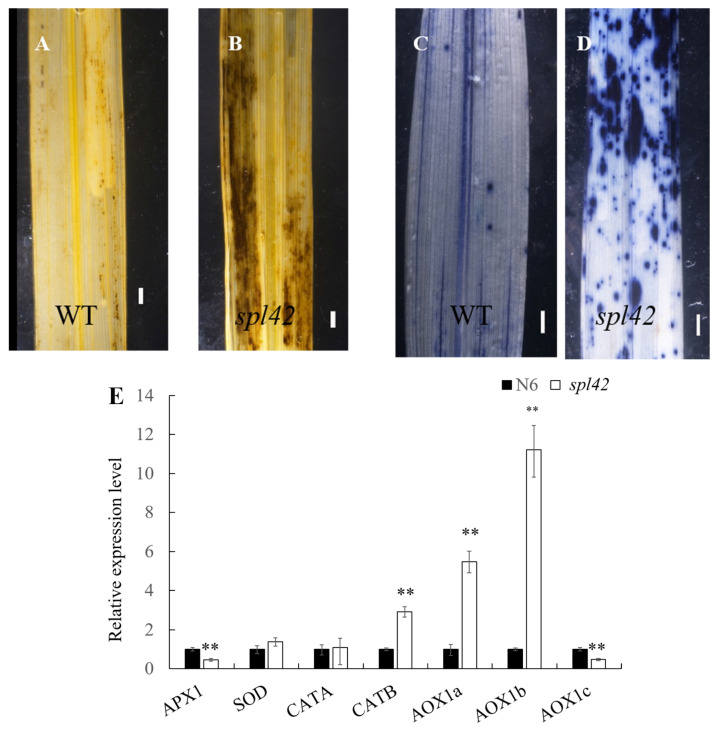
Comparison of reactive oxygen species (ROS) activity between wild-type and *spl42* mutants and expression analysis of genes related to ROS scavenging. (**A**,**B**) Hydrogen peroxide (H_2_O_2_) was visualized by diaminobenzidine (DAB) staining. Scale Bar = 0.1 cm. (**C**,**D**) Superoxide anion (O^2−^) was visualized by nitro-blue tetrazolium (NBT) staining. Scale Bar = 0.1 cm. (**E**) qRT-PCR analysis of the genes related to ROS scavenging systems. Error bars represent ± SD (*n* = 3). Asterisks indicate a significant difference between NingJing6(N6) and the *spl42* plants by Student’s *t*-test, ** *p* < 0.01.

Previous studies demonstrated that leaf spot symptoms were associated with a hypersensitive response (HR), accompanied by the accumulation of ROS [13]. Diaminobenzidine (DAB) could react with hydrogen peroxide (H_2_O_2_) to form a dark brown polymerization product, while nitro-blue tetrazolium (NBT) could be restored to a dark blue insoluble substance by O^2-^ [10,11]. Therefore, we used DAB and NBT staining to detect the ROS accumulation level in the leaves of wild type and *spl42* mutant seedlings. The DAB staining showed that the H_2_O_2_ in the mutant was obviously accumulated (Figure 3A,B). Similarly, the NBT staining indicated that the mutant had a higher level of superoxide anions (O^2−^) accumulation compared with the wild type (Figure 3C,D). In addition, qRT-PCR was performed to analyze the expression of some genes involved in the ROS scavenging system, including *APX1* and *APX2* (encoding two isoforms of ascorbate peroxidase genes), *SOD-A* (encoding superoxide dismutase gene), *CATA* and *CATB* (encoding two isoforms of catalase genes), *AOX1a*, *AOX1b*, and *AOX1c* (encoding three isoforms of alternative oxidase gene). Our results showed that the expression levels of *CATB*, *AOX1a,* and *AOX1b* were significantly increased compared to the wild type (Figure 3E), indicating that the ROS levels in the *spl42* mutant was much higher than those in the wild type. The up-regulated expression of genes related to the antioxidant system might be a protective mechanism for the normal plant growth against physiological stress conditions.

**Figure A1:**
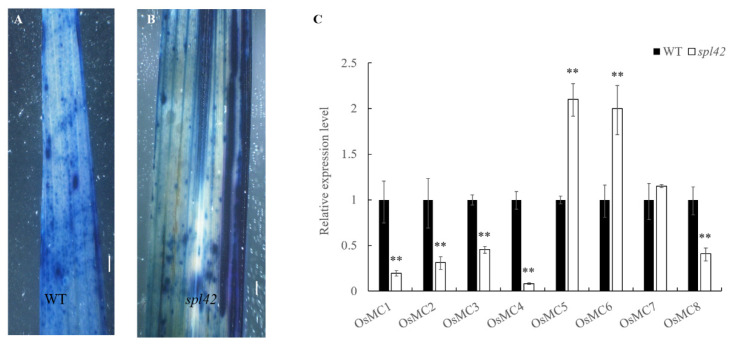
Trypan blue staining experiment and meta caspase gene family expression analysis. (**A**,**B**) Trypan blue staining in wild type (WT) and mutant *spl42* leaves. Scale bars = 0.1 cm. (**C**) qRT-PCR analysis for meta caspase family genes. Error bars represent ± SD (*n* = 3). Asterisks indicate a significant difference between WT and the *spl42* plants by Student’s *t*-test, ** *p* < 0.01.

At the same time, plant leaves were stained with trypan blue for the detected cell activity. The results showed that the *spl42* mutant displayed a lot of lesion spots, while the wild type did not show any lesions (Figure A1A,B). The gene expression associated with programmed cell death indicated that two metacaspase gene family members were significantly upregulated (Figure A1C). Correspondingly, the content of relevant reactive oxygen removal enzymes and reactive oxygen-related substances were mostly elevated in the *spl42* mutant (Table A2).

### 2.4. Elevated Expression of Defense Response Genes

**Figure 4:**
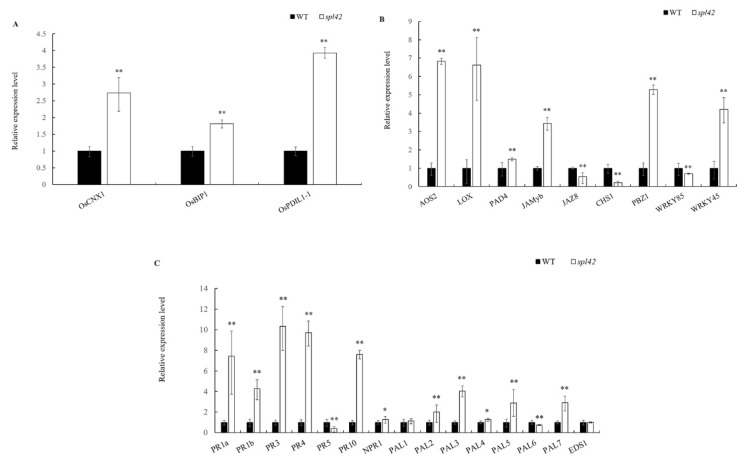
Expression analysis of defense-related gene families in wild type (WT) and *spl42*. (**A**) Endoplasmic reticulum chaperone gene qRT-PCR analysis. (**B**) qRT-PCR analysis of JA signaling genes. (**C**) qRT-PCR analysis of SA signaling genes. Error bars represent ± SD (*n* = 3). Asterisks indicate a significant difference between the WT and *spl42* plants by Student’s *t*-test, ** *p* < 0.01; * *p* < 0.05.

The activation of defense responses and an enhanced disease resistance have been reported in a number of lesion-mimic mutants [26,27]. We first analyzed the expression levels of endoplasmic reticulum (ER) chaperone genes consisting of *OsCNX1* (encoding cofactor for nitrate reductase and xanthine dehydrogenase1), *OsBip1* (encoding an ER molecular chaperone protein), and *OsPDIL1-1* (a marker indicator of unfolded protein accumulation in the ER caused by ROS accumulation) [28]. The expression levels of these genes in the *spl42* mutant increased by 1.7-fold, 0.8-fold, and 2.9-fold, respectively, compared with the wild type (Figure 4A).

We further analyzed the changes in the expression of the defense-related genes involved in jasmonic acid (JA) and salicylic acid (SA) signaling and/or biosynthesis. Compared with the wild type, the expression levels of JA signaling genes including *AOS2* (encoding propylene oxide synthase), *LOX* (encoding lipoxygenase), *PAD4* (encoding plant anti-toxin protein), *JAMyb* (encoding R2R3-type MYB transcription factor), *PBZ1* (encoding disease-course related protein), and *WRKY45* (encoding WRKY transcription factor) were significantly elevated in *spl42*, whereas the expression of *JAZ8* (encoding a jasmonate ZIM structural domain protein), *CHS1* (encoding Chalcone synthetase1), and *WRKY85* (encoding a WRKY transcription factor) was significantly lower (Figure 4B). In contrast, most SA signaling genes *PR1a*, *PR1b*, *PR3*, *PR4*, and *PR10* (encoding pathogenesis-related proteins), *NPR1* (non-expressor of pathogenesis-related genes 1), and *PAL2*, *PAL3*, *PAL4*, *PAL5*, and *PAL7* (encoding phenylalanine deaminase genes) were highly up-expressed in *spl42* compared with the wild type (Figure 4C).

### 2.5. Map-Based Cloning of the SPL42 Gene

**Figure 5:**
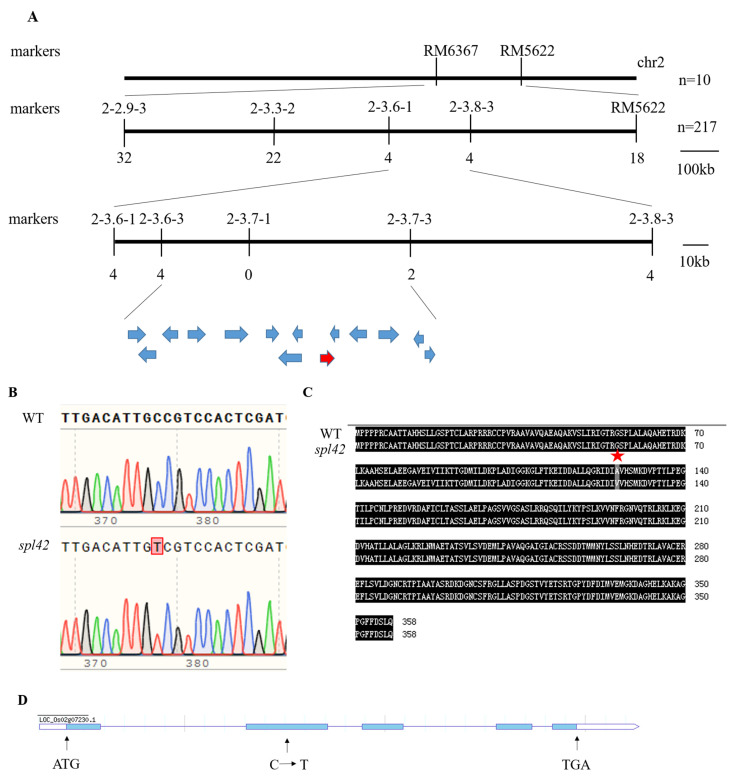
Map-based cloning of the *SPL42* gene. (**A**) The *SPL42* locus was delimited to a 123 kb genomic region between markers 2-3.6-3 and 2-3.7-3 on the short arm of chromosome 2. Fourteen open reading frames (indicating by arrow) were predicted in the region. Numbers below the chromosome indicate the numbers of recombinant individuals in gene mapping. (**B**) Sequencing results of the wild type (WT) and the *spl42* mutant. The mutated nucleotide was boxed. (**C**) Amino acid sequence alignment of the wild type and *spl42* mutant. A star indicates the putative amino acid change between the wild type and *spl42* mutant. (**D**) *SPL42* gene structure. ATG represent the start codon and TAG is stop codon, and the blue boxes represent exons, while the lines between the blue boxes represent introns. The single base substitution of C-T (arrow) causes amino acid changes.

An F2 population derived from the cross between the *spl42* mutant and N22 was used to map the *SPL42* locus. The segregation in the F2 population was 754 plants with normal (wild-type) leaves, and 217 with reddish-brown spotted leaves, corresponding to a 3:1 ratio (754:217, χ2 = 1.89 < χ2 _0.05,1_ = 3.84; *p* = 0.1692 > 0.05), indicating that the spotted leaf phenotype in *spl42* was controlled by a single recessive-nuclear gene. The *SPL42* locus was initially mapped by 10 recessive reddish-brown spotted leaf individuals between the markers RM6357 and RM5622 on the short arm of chromosome 2 (Figure 5A). Based on an analysis of 217 plants with a recessive (mutated) phenotype, *SPL42* was delimited to a 123 kb genomic region between markers 2-3.6-3 and 2-3.7-3 containing fourteen open reading frames (ORFs) (Figure 5A). The specific data and information of the fourteen open reading frames were listed in the table through a website prediction (http://plants.ensembl.org/info/about/collaborations/gramene.html, accessed on 15 December 2022) (Table A3). Sequencing analysis showed that only the second exon of ORF9 (*Os02g0168800*) had a single-base C to T substitution, resulting in the conversion of alanine to valine (Figure 5B–D). The sequencing of other thirteen candidate genes revealed no base changes between the *spl42* mutant and its wild type. Taken together, these results indicated that *Os02g0168800* was the *SPL42* candidate gene.

**Figure 6:**
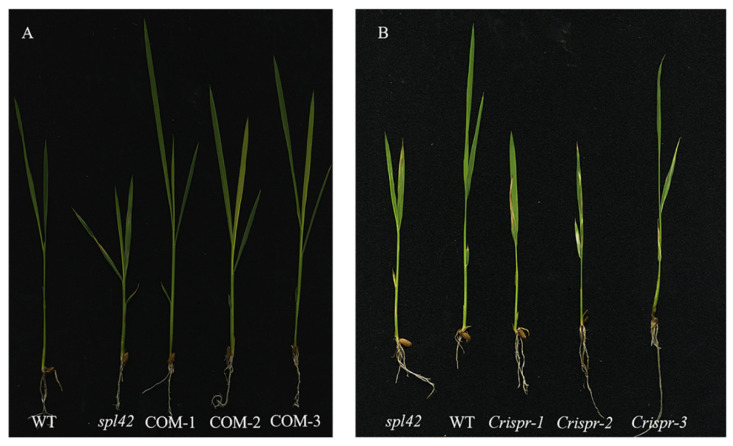
*SPL42* complementation and knockout transgene verification. (**A**) Phenotypes of wild type, *spl42* mutant, and transgenic complementary plants at the four-leaf stage. (**B**) Phenotypes of *spl42* mutant, wild type, and three knockout mutants Crispr-1/2/3.

The genomic sequence of the *Os02g0168800* gene is 2993 bp and contains five exons and four introns. To further confirm that the single-base substitution in *Os02g0168800* was responsible for the spotted leaf phenotype of the *spl42*, we generated *SPL42* transgenic complementary plants in the *spl42* mutant background. *SPL42* transgenic complementary plants recovered the green leaves phenotype similar to that of the wild type at the seedling stage (Figure 6A). In order to further clarify the function of *SPL42*, a knockout mutant of *SPL42* was generated by CRISPR/Cas9 technology in the NingJing6 background, which showing a spotted leaf phenotype similar to that of the mutant (Figure 6B). These results demonstrated that *SPL42* was a positive regulator of the leaf development in rice.

### 2.6. Bioinformatics Analysis of SPL42

**Figure 7:**
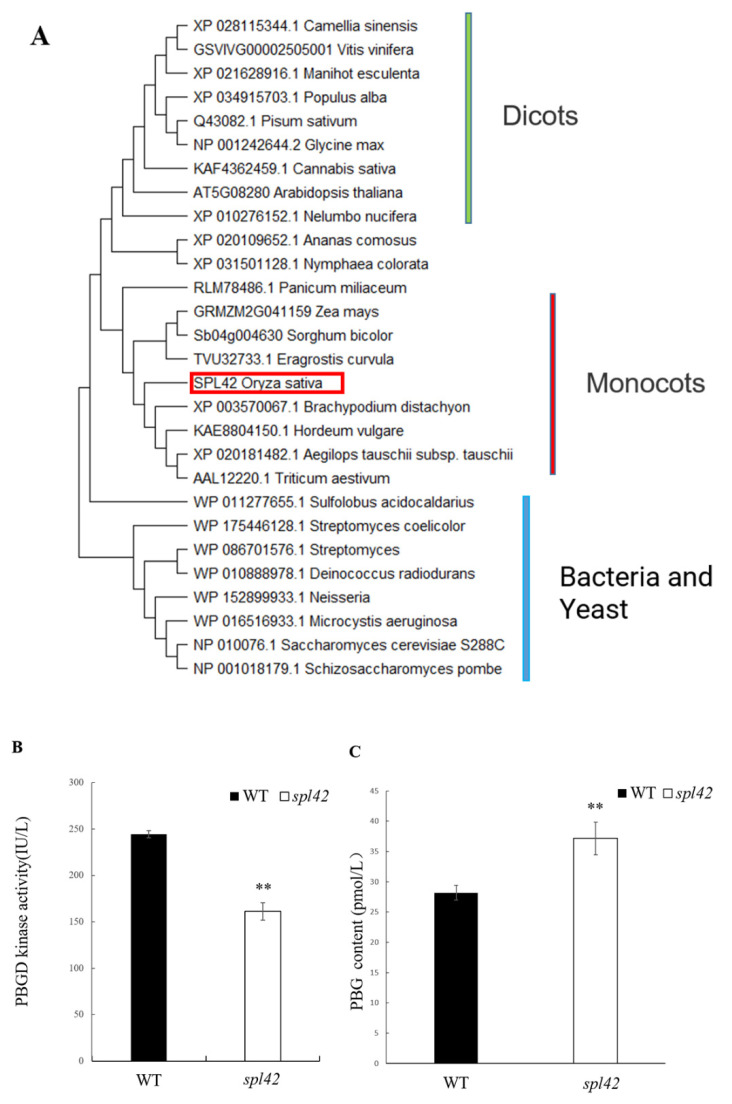
Phylogenetic tree analysis of *SPL42* and enzyme properties of porphobilinogen deaminase (PBGD). (**A**) Phylogenetic tree analysis of *SPL42* protein (boxed) with homologous proteins. The rooted tree using percentage identities is based on a multiple sequence alignment generated with the program MEGA-X. Scale represents percentage substitution per site. (**B**) Enzyme activity analysis of porphobilinogen deaminase (PBGD) in wild type (WT) and *spl42* mutants. Error bars represent ± SD (*n* = 3). (**C**) porphobilinogen (PBG) content of the precursor of enzyme reactions in wild type and *spl42* mutants. Error bars represent + SD (*n* = 3). Asterisks indicate a significant difference between WT and the *spl42* plants by Student’s *t*-test, ** *p* < 0.01.

According to the functional prediction of the *SPL42* protein sequence on the NCBI website, *SPL42* encodes a PBGD, which is a key enzyme in the chlorophyll synthesis pathway. The homologues of *SPL42* were previously reported in Arabidopsis and maize species, both of which showed a lesion mutant phenotype once their normal functions were disrupted [7,8]. We compared the *SPL42* protein sequences with other proteins from different species via the NCBI website and constructed phylogenetic trees to study the evolutionary relationships between *SPL42* and its homologues (Figure 7A). The results showed that *SPL42* was 93%, 91%, 90%, and 88% homologous to its counterparts in *Eragrostis curvula* (TVU32733.1), maize (GRMZM2G041159), sorghum (Sb04g004630), and proso millet (RLM78486.1), respectively. The phylogenetic tree was divided into three branches, including monocots, dicots, bacteria, and yeast, and *SPL42* was highly conserved among monocots. Furthermore, we found only one purine choline deaminase in the rice genome, suggesting that *spl42* was a weak mutation in a single copy gene.

Considering that *SPL42* encoded a PBGD, the enzyme activity of *SPL42* and the content of the precursors for enzymatic reactions were measured. Our results showed that the enzyme activity of the *spl42* mutant was decreased significantly, whereas the content of the precursors for enzymatic reactions was increased significantly (Figure 7B,C). These results indicated that the single base change in *spl42* inhibited PBGD enzyme activity and subsequently resulted in the decrease in the chlorophyll content.

### 2.7. Expression Analysis of SPL42

**Figure 8:**
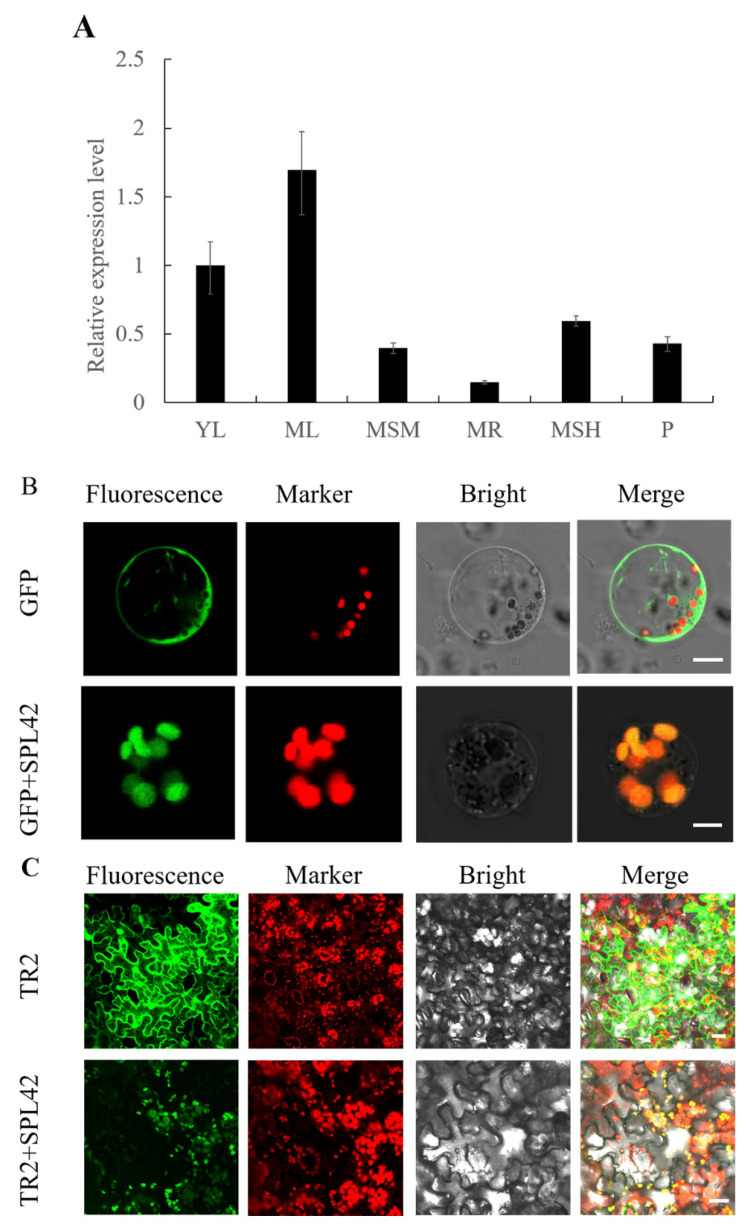
Expression pattern and subcellular localization of *SPL42*. (**A**) Relative expression levels of *SPL42* in the different tissues. YL: young leaf at the four-leaf stage; ML: mature leaf; MSM: mature stem; MR: mature root; MSH: mature sheath; P: panicle. Error bars represent + SD (*n* = 3). (**B**) Subcellular localization of SPL42 in rice protoplasts. (**C**) Subcellular localization of SPL42 in tobacco leaves. TR2 indicates SPL42-GFP fusion vector was driven by an octopine type Ti-plasmid right T-DNA gene 2′ promoter (TR 2). Signals from GFP fluorescence, chlorophyll autofluorescence, bright field, and merged images were shown. Scale Bar = 2 μm in (**B**) and 20 μm in (**C**).

The *SPL42* transcripts were detected in the roots, stems, young leaves, mature leaves, leaf sheaths, and developing panicles, indicating that *SPL42* was a constitutively expressed gene (Figure 8A). The expression level of *SPL42* was the highest in mature leaves and relatively low in root tissues, consistent with its role in photosynthesis.

We use the online tool ChloroP (http://www.cbs.dtu.dk/services/ChloroP/, accessed on 15 December 2022) to predict the *SPL42* protein and found that its N-terminal harbored a chloroplast transit peptide, suggesting that *SPL42* may locate in the chloroplast. To confirm the subcellular localization, the full-length *SPL42* cDNA was amplified from wild-type cDNA, and was inserted in the *PAN580* plasmid and transformed into rice protoplasts. Confocal microscopy showed that the green fluorescence of the *GFP-SPL42* fusion protein colocalized with the autofluorescence signal of chlorophyll, indicating that *SPL42* was indeed located in chloroplasts (Figure 8B). Further, the *SPL42* localization to chloroplasts was verified by the transient expression of the *TR2- SPL42* fusion protein in tobacco leaves (Figure 8C).

### 2.8. SPL42 Interacts with OsMORF8-1 and OsMORF8-2 and Is Involved in RNA Editing

**Figure A2:**
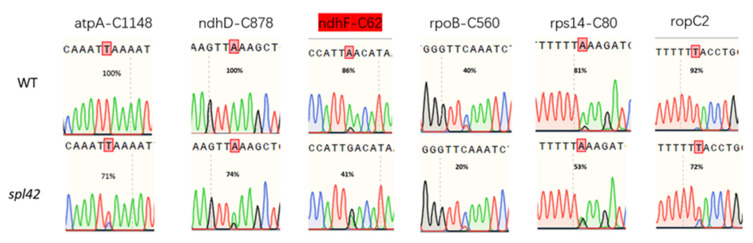
Changes in RNA editing efficiency in chloroplasts of the wild type and the *spl42* mutant. The red box represents the only RNA editing site that changes amino acid of encoding protein.

RNA editing is a correction mechanism for pyrimidine-cytidine mutations in plant organelle genomes. It was previously reported that some chlorophyll synthesis-related enzymes, including protoporphyrinogen IX oxidase 1 (PPO1) and HEMC (an Arabidopsis homologous protein of SPL42), participate in RNA editing in Arabidopsis [29,30]. Since SPL42 is localized to chloroplasts, we examined 27 reported RNA editing sites in rice chloroplasts, as reported previously [21,31]. The results showed that six loci were significantly different, including atpA-C1148, ndhD-C878, ndhF-C62, rpoB-C560, rps14-C80, and ropC2 (Figure A2). However, only one of them, i.e., ndhF-C62, caused an amino acid change in the encoding protein. These results suggested that SPL42, similar to HEMC, not only affected the chlorophyll synthesis but also participated in RNA editing in rice.

**Figure 9:**
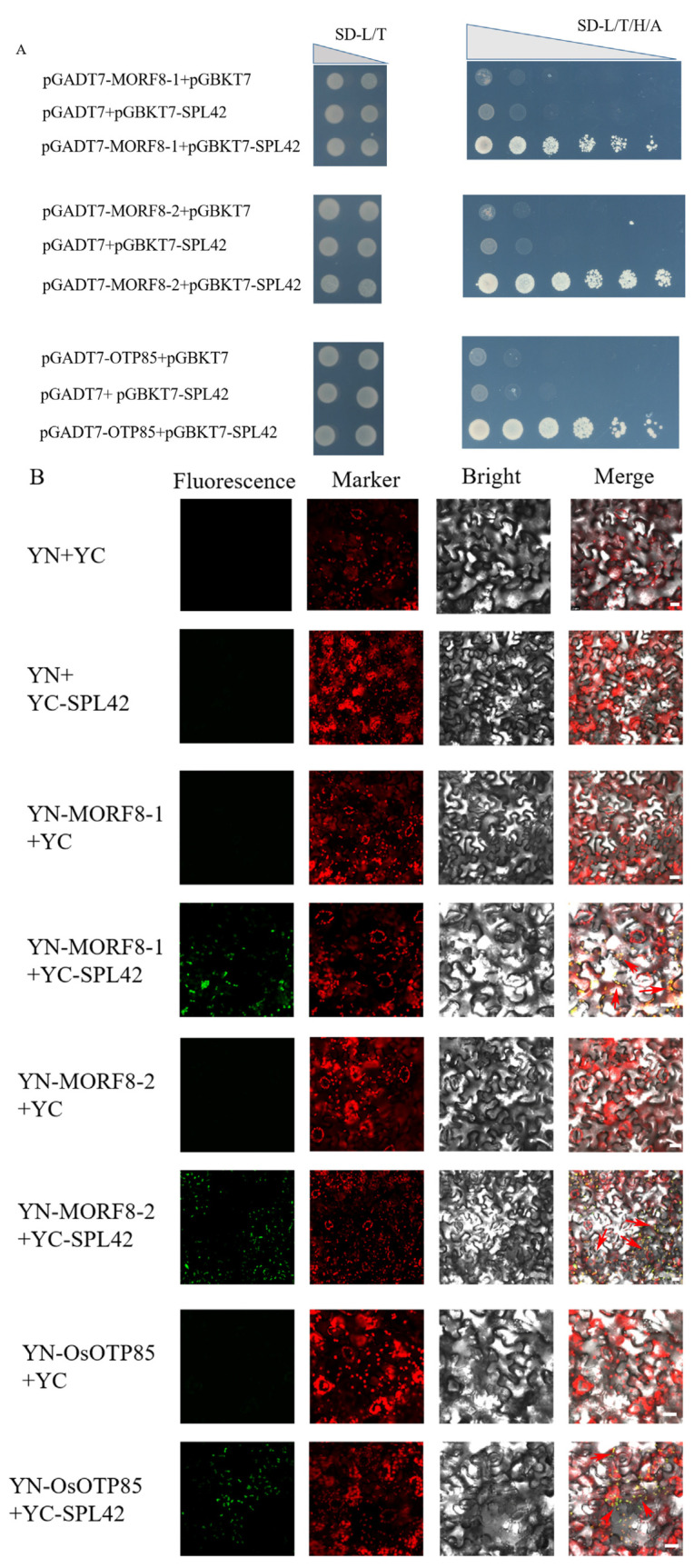
SPL42 physically interacts with OsMORF8-1 and OsMORF8-2. (**A**) SPL42 physically interacts with OsMORF8-1 and OsMORF8-2 in yeast. The indicated construct pairs were co-transformed into yeast strain AH109. Interactions between bait and prey were examined on control medium 2 (SD/-Leu/-Trp) and selective medium 4 (SD/-Leu/-Trp/-His/-Ade). AD: activation domain; BD: binding domain. (**B**) Bimolecular fluorescence complementation assay showing the interactions between SPL42 and OsMORF8-1 as well as OsMORF8-2 in tobacco leaf epidermal cells. Signals from GFP fluorescence, chlorophyll autofluorescence, bright field, and merged images are shown. The arrows indicate signal areas with a strong interaction. Scale Bar = 20 μm.

The multiple organelle RNA editing factor (MORF)/RNA-editing factor interacting protein (RIP) extensively influenced the RNA editing sites in mitochondria and plastids [24,25]. In rice, there are a total of seven MORFs, among which OsMORF1(Os11g11020) and OsMORF3(Os03g38490) proteins are localized in mitochondria, and OsMORF2-1(Os04g51280), OsMORF2-2(Os06g02600), and OsMORF9(Os08g04450) proteins are localized in chloroplasts. However, OsMORF8-1(Os09g04670) and OsMORF8-2(Os09g33480) are a mitochondrial and chloroplast dual localization [21]. We thus investigated whether SPL42 interacted with MORFs. A yeast two-hybrid (Y2H) experiment demonstrated that SPL42 physically interacted with OsMORF8-1 and OsMORF8-2 (Figure 9A). A bimolecular fluorescence complementation (BiFC) assay revealed that SPL42, OsMORF8-1, and OsMORF8-2 all localized to overlapping cellular compartments and interacted with each other to generate a GFP fluorescence in the nuclei of tobacco leaf cells (Figure 9B).

Previous studies showed that HEMC interacted with the PPR-protein OTP85 to influence RNA editing in Arabidopsis [24,30]. The OTP85 homolog in rice, i.e., OsOTP85 (Os03g0795200), also showed the interaction with SPL42 by Y2H and BiFC experiments (Figure 9A,B). These results demonstrated that the SPL42 was involved in RNA editing through an association with MORFs and PPR(s).

### 2.9. RNA-Sequencing Analysis

**Figure 10:**
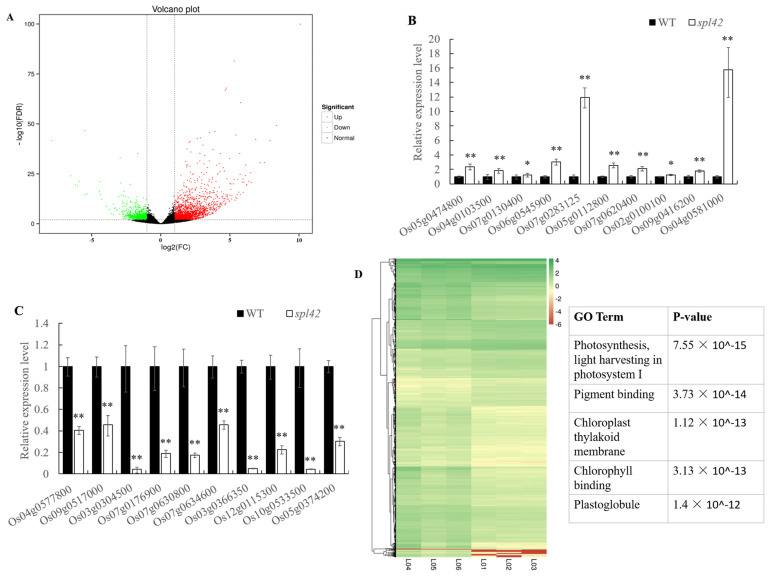
RNA-sequencing related data analysis of the wild type (WT) and the *spl42* mutant. (**A**) Distribution of volcano plot of wild type and *spl42* mutant. Volcano plot shows the differential expression of genes in accordance with fold difference and significant levels, and the criteria for significant differentially expressed gene screening were: |log^2^Ratio| ≥ 1 and q-value ≤ 0.05. (**B**,**C**) qRT-PCR analysis of 10 up-regulated and 10 down-regulated genes which are selected randomly from RNA-sequencing results. Asterisks indicate a significant difference between WT and the *spl42* plants by Student’s *t*-test, ** *p* < 0.01; * *p* < 0.05. (**D**) Cluster analysis of differentially expressed genes in wild type and *spl42* mutants.

**Figure A3:**
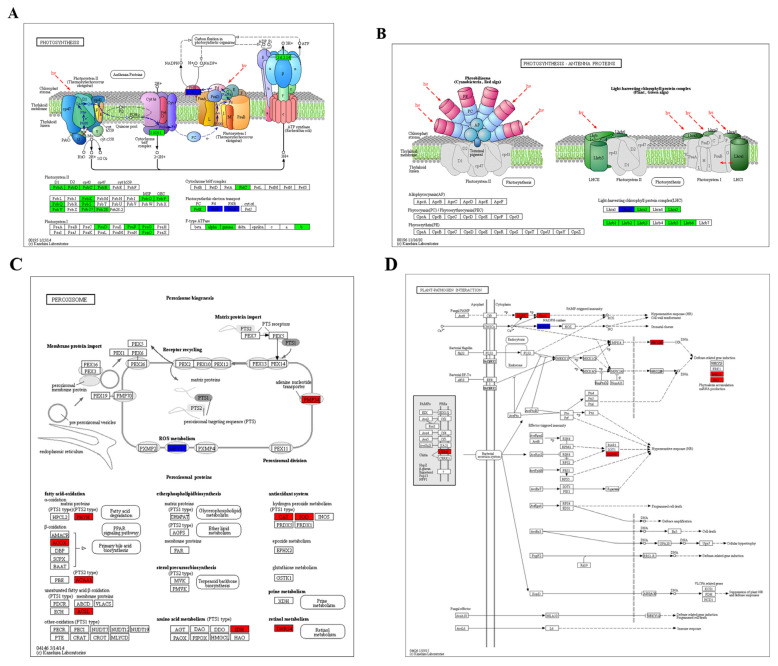
Differential map of genes in different pathways between wild type and *spl42* mutant. (**A**) Differential genes in photosynthesis pathway. (**B**) Differential gene of photosynthesis-antenna protein. (**C**) Differential genes of the peroxisome pathway. (**D**) Differential genes for the interaction between plants and pathogens.

To comprehensively understand the effect of the *spl42* mutant on the gene expression, we selected the leaves of wild-type and *spl42* mutant plants at the four-leaf stage for RNA-seq analysis. A summary of the transcriptome sequencing data can be found in the attached Figure A3. It was noteworthy that the RNA-seq results were consistent with the aforementioned RT-PCR analyses. A total of 2772 differentially expressed genes (DEG) were detected, of which 1777 genes were up-regulated and 995 genes were down-regulated in the *spl42* mutant (Figure 10A). In order to further verify the reliability of RNA-seq data, we randomly selected 10 up-regulated and 10 down-regulated genes for qRT-PCR analysis. As predicted, the results of qRT-PCR were generally consistent with those of RNA-seq analysis (Figure 10B,C). The clustering analysis of differentially expressed genes showed that most significant gene expression differences were concentrated in the light harvesting and pigment synthesis pathways in photosystem I (Figure 10D). It was reasonable that the expression changes in these genes in *spl42* led to the failure of the photosynthesis pathway. Furthermore, we found that the expression levels of ten genes related to porphyrin, the chlorophyll metabolism, and the carotenoid biosynthesis pathway, twenty-five genes related to the photosynthesis pathway were down-regulated, whereas seventeen genes related to peroxisome (a primary organelle associated with ROS production) were significantly up-regulated (Figure A3). Moreover, our results of the RNA-seq indicated that most of the genes associated with a disease resistance were up-regulated in *spl42*. Collectively, the RNA-seq analysis revealed that the *spl42* might promote a ROS production by affecting the efficiency of chlorophyll synthesis and photosynthesis.

## 3. Discussion

### 3.1. spl42 Is a Temperature-Sensitive Mutant

The normal formation and development of chloroplasts play a decisive role in breeding high-yield crops. The discovery of leaf color mutants provides an ideal material for studying the chloroplast metabolism, development, and photosynthesis, as well as molecular mechanisms of chlorophyll biosynthesis [32]. Among the reported mutants of rice leaf spots, the lesion-like phenotype is largely affected by environmental conditions such as the temperature, light, and humidity [33]. For example, the formation of diseased spots in the *spl7* mutant was induced by a high temperature and ultraviolet radiation [14]. Conversely, leaf necrosis was inhibited by a high temperature and long day in *OsLSD1* antisense plants [33]. In this study, we identified the high-temperature inducible mutant *spl42*, which is similar to *spl7*. The phenotype of *spl42* was the most obvious under high temperature conditions (30 °C) (Figure 1K) and, correspondingly, the chlorophyll content was decreased significantly (Figure 1L). In addition, when *spl42* was growth in the field of Hainan, China (a natural low temperature condition), the leaf spot formation was later than its wild type (data not shown). The lesion spots in *spl42* also resulted in a significant decrease in the photosynthetic-related parameters such as the light quantum yield and photosynthetic rate. Moreover, *spl42* exhibited other abnormal phenotypes, including the reductions in the plant height, panicle length, flag leaf length, and seed setting rate compared with the wild type (Table A1). These results suggest that *SPL42* plays a pleotropic role in rice.

### 3.2. Disruption of Chlorophyll Synthesis Pathway Leads to the Production of Abnormal Chloroplast

Imbalances in metabolic pathways can lead to the accumulation of intermediate metabolites, thereby inducing autoimmunity and cell death. For example, *SPL18* encodes an acyltransferase [34], and *OsSSI2* encodes a fatty acid desaturase. Mutations in both genes produced a necrotic plaque phenotype on the leaves [35]. Likewise, the mutation of the protoporphyrogen III oxidase gene *RLIN1* in the tetrapyrrole biosynthesis pathway led to a leaf spot formation and programmed cell death [36,37]. In our study, the defect of *SPL42* caused a significant reduction in not only enzyme activity but also the precursor deposition of the PBGD enzyme reactions (i.e., porphobilinogen) in rice plants (Figure 7B,C). Since porphobilinogen is one of the tetrapyrrole, we hypothesized that the tetrapyrrole intermediates in *spl42* were photoactivated and oxidized by light, resulting in the accumulation of reactive oxygen intermediates and photodynamic destruction. Moreover, the TEM analysis showed that there were two types of chloroplasts in *spl42*: one kind of chloroplasts was bigger in size than the wild type, and its thylakoid arrangement was relatively loose, while the chloroplast of the other one was broken and degraded and could not produce the complete thylakoid lamella structure (Figure 2A–F). These results suggested that the phenotype of leaf color mutants was closely related to the development of chloroplast and chlorophyll biosynthesis. RNA-sequencing results further confirmed the down-regulation of porphyrin, the chlorophyll metabolism, and carotenoid biosynthesis genes in *spl42*, consistent with our results that chloroplast numbers are crucial for ensuring the chloroplast’s structure.

### 3.3. The Disorder of ROS Scavenging System May Result in the Enhancement of Defense Response in spl42

Maintaining the homeostasis of ROS is pivotal for normal plant growth and defense response. In many spotted leaf mutants, the balance of ROS was broken, leading to the disturbance of a plant development [15,16,17]. Under light conditions, chloroplasts in plant cells are a major source of ROS and have been shown to control programmed cell death in response to ROS [38]. Chloroplast damage is caused by oxidative bursts that lead to the excessive deposition of material, such as plastoglobules and callose, in the chloroplast [38]. In our study, the levels of ROS were abnormal in the *spl42* mutant. DAB and NBT staining experiments confirmed the excessive accumulation of H_2_O_2_ and O^2-^ in the mutant (Figure 3A,B). In addition, the expression of SOD, POD, and MDA of the ROS scavenging system were mostly up-regulated in *spl42* (Table A2). These results suggested that the disruption of ROS homeostasis might be one of the reasons for the formation of the spotted leaf. More than 80% of the reported spotted leaf mutants showed an enhanced resistance to rice blast or bacterial blight [11,39,40,41,42]. For example, the *spl40* showed a higher resistance to bacterial blight strains PXO79, PXO145, C5, and OS225 than its wild type [43]. The resistance of the *spl35* to grisea and bacterial strains CH43, CH680, and PXO61 was also significantly enhanced [27]. The RNA-Seq analysis revealed that the genes related to the disease resistance were upregulated in *spl42*, which was consistent with the changes in the expression levels of the defense genes related to the SA and JA pathways (Figure 4A–C).

### 3.4. spl42 Is a Temperature-Sensitive Mutant

Previous reports indicated that Arabidopsis HEMC was involved in RNA editing by interacting with MORF family proteins, and PPO1 is involved in RNA editing as an enzyme of the chlorophyll synthesis pathway [29,30]. We examined 27 reported RNA editing genes in rice chloroplasts. Of these, six differed in nucleotides, including *atpA-C1148*, *ndhD-C878*, *ndhF-C62*, *rpoB-C560*, *rps14-C80*, and *ropC2,* but only one of these *(ndhF-C62)* underwent a non-synonymous base change (Figure A2). In addition, yeast two-hybrid experiments and bimolecular fluorescence complementation experiments showed that SPL42 interacted with OsMORF8-1 and OsMORF8-2, as well as OsOTP85 (Figure 9A,B). Thus, SPL42 might be involved in RNA editing with OsMORFs and PPR protein(s). Although a model was proposed to decipher the association of MORFs, PPRs, and HEMC [30], the detailed molecular mechanism between SPL42 and RNA editing needs to be uncover in future.

## 4. Materials and Methods

### 4.1. Plant Materials and Growth Conditions

The rice *spl42* mutant was isolated from rice cultivar (cv.) NingJing6. An F2 population derived from a cross between *spl42* and N22 was used for the genetic analysis. Wild-type and *spl42* mutant plants were grown in Nanjing (Jiangsu province) and Lingshui (Hainan province) under natural conditions. For temperature-sensitive treatments, plants in the growth chamber (GXM-258B, Ningbo) were treated with a 16 h light/8 h darkness photoperiod at a constant temperature of 20 °C, 25 °C, or 30 °C, respectively [44]. The thousand-grain weight, seed length, and width were examined by a seed phenotyping system (SC-G automatic test seed analysis software, Hangzhou). At the maturity of the wild type and *spl42*, the plant height, number of tillers, spike length, and leaf length and width were investigated. At seed harvest, agronomic traits such as the number of branches, seed set, number of grains per spike, grain length, grain width, and thousand-grain weight were investigated. The photosynthetic rate and other photosynthetic indicators of the wild type and *spl42* mutant were measured at the maximum tillering stage using a portable photosynthesizer (Li6400XT, LI-COR, USA) and an ultra-portable modulated chlorophyll fluorometer (MINI-PAM, WALZ, Germany). Twenty plants were surveyed for each trait and the mean was taken.

### 4.2. Measurement of Chlorophyll Contents

About 0.03 g of fresh leaves of the wild type and *spl42* mutant plants at the four-leaf stage were collected to determine the chlorophyll contents. The cut leaves were soaked in 95% ethanol and left out of the light for 48 h. After centrifugation, the supernatant was collected and the optical density at 665, 649, and 470 nm was measured by using a spectrophotometer. Three replicates of different samples were set up. The contents of chlorophyll a, chlorophyll b, and carotenoids were calculated according to the method described previously [45].

### 4.3. TEM Analysis

For TEM analysis, normal green and spotted leaves from the fourth leaf of 2-week-old wild-type and mutant plants grown in the field were cut into small pieces of 0.5 cm. The detailed method was described in the previous studies [44] with minor modifications. The leaves were fixed with 2.5% glutaraldehyde solution and vacuumed until fully sinking to the bottom, then rinsed and incubated in 1% OsO4 (PH = 7.2) overnight at 4 °C. After staining with uranyl acetate, the tissues were further dehydrated in an ethanol series and then embedded in Spurr’s medium prior to ultrathin sectioning. Finally, the samples were stained again and observed with a Hitachi H-7650 transmission electron microscope (HITACHI, Tokyo, Japan).

### 4.4. DAB and NBT Staining

DAB can react with H_2_O_2_ to form a dark brown polymerization product, while NBT can be restored to a dark blue insoluble substance by O^2−^. Therefore, the detection of O^2−^ and H_2_O_2_ was conducted by staining with NBT and DAB. We selected the third leaves of the *spl42* mutant and wild type at the four-leaf stage to determine the accumulation level of reactive oxygen species. The third leaves were soaked in the prepared dye, then vacuumed and stained overnight at 37 °C in darkness. The next day, the tissues were decolorized with 95% ethanol until the color no longer changed.

### 4.5. Trypan Blue Staining Test

Trypan blue, a staining reagent for testing the cell membrane integrity, was used to stain the third leaf of the four-leaf stage of wild type and mutant *spl42* leaves grown normally under field conditions. Dead cells were stained dark blue and normal cells were barely stained, as previously reported [28].

### 4.6. Determination of Reactive Oxygen Scavenger Enzyme Activity and Related Indexes

In order to study the accumulation of ROS in the mutant *spl42*, the third leaves of the *spl42* and wild type were selected under field conditions, and the enzymatic activities of CAT, SOD, and POD, as well as the content of H_2_O_2_ and MDA, were measured using kits from Jiancheng Biological Co., Ltd., Nanjing, Jiangsu, China (http://www.njjcbio.com/, accessed on 15 December 2022).

### 4.7. Map-Based Cloning of SPL42

For gene mapping, F1 was obtained by hybridization with the *spl42* mutant as the female parent and indica cultivar N22 as the male parent. Then, F1 plants were self-crossed to obtain F2 generation. The F2 populations were used for mapping the *SPL42* gene. Ten *spl42*-like individual DNA of the F2 population were selected for linkage analysis of the whole genome. Then, additional individuals were used to narrow down the *spl42* locus. Primers for a fine localization were listed in Table A4. An SSR primer amplification system (10 µL): template DNA (20 ng/µL) 1 µL, primer (2 mmol/L) 1 µL, mix 4 µL, ddH_2_O 4 µL. The amplification procedure was as follows: pre-denaturation at 94 °C for 10 s, denaturation at 98 °C for 30 s, annealing at 58 °C for 30 s, and extension at 72 °C for 40 s. The denaturation–annealing–extension steps were carried out for 34 cycles, and the last step was 72 °C for 10 min and storing at 4 °C.

### 4.8. Transgene Constructs and Plant Transformation

For the complementation experiments, a fragment including the promoter region (2 kb) and full-length cDNA of *SPL42* in the wild type was amplified with the HB-F and HB-R prime pairs. Then, the correct PCR product was inserted to the pCUBi1390-Flag vector. The resultant recombinant construct *pCUbi1390-SPL42* was introduced into the *spl42* mutant by *Agrobacterium*-mediated transformation using the *Agrobacterium tumefaciens* strain EHA105 [46]. For the construction of transgenic knockout, a 20 bp fragment (CCTTGCCACCTATATCTGCC) targeted to *SPL42* was designed using the CRISPR-Cas9 website tool (http://cbi.hzau.edu.cn/cgi-bin/CRISPR, accessed on 15 December 2022). The targeted fragment was inserted into the CRISPR/Cas9 binary vector pCAMBIA1305.1 carrying the CaMV35S promoter. The sequenced construct was introduced into the Japonica rice variety NingJing6. The primers used for vector construction were listed in Table A4.

### 4.9. Enzyme Activity Assay In Vivo

About 0.5 g of fresh leaves from the wild-type and the *spl42* mutant seedlings were weighed and mixed with 10 mL of phosphate buffer (PBS, pH = 7.4) after grinding in liquid nitrogen. Then, after ultrasonic crushing for 5–10 min, the supernatant was aspirated by centrifugation and prepared for the determination of the enzyme activity. The test method was according to the manual of plant CAT, SOD, PAD, and PBGD ELISA (enzyme-linked immunosorbent assay) kits (Shanghai Zhen Ke Biological Tecchnology Co., Ltd., http://www.shzkbio.com/, accessed on 15 December 2022).

### 4.10. RNA Preparation and Reverse Transcription Quantitative PCR (RT-qPCR)

Total RNA was extracted from the wild type and the *spl42* mutant at different leaf stages (the three-leaf to four-leaf stage) or different tissues. The detailed extraction protocol followed the instruction of an RNA Prep Pure Plant kit (TIANGEN Biotech, Beijing, China, http://www.tiangen.com/en/, accessed on 15 December 2022). The total RNA (2 μg) was used for a single-strand cDNA synthesis using Superiorscript Reverse Transcriptase (Enzynomics, http://www.enzynomics.com, accessed on 15 December 2022) and oligo(dT) primers. Three technical replicates on three biological replicates were carried out for each sample. qRT-PCR was conducted using PikoReal real-time PCR (ThermoFisher Scientific, https://www.thermofisher.com, accessed on 15 December 2022) with SYBR Green Premix Ex TaqII (Takara, https://www.takarabio.com, accessed on 15 December 2022) according to the manufacturer’s instructions. The primers for the qRT-PCR were listed in Table A4, and the *UBQ5* (ubiquitin5) was used as an internal control to normalize the RT-qPCR results. Data were analyzed and processed using the 2^-ΔΔCT^ method [44,47].

### 4.11. Validation of Intrachloroplast RNA Editing

The total RNA was isolated using the RNAprep pure Plant Kit (Tengen Biochemistry Ltd.) and first-strand cDNA was synthesized using the PrimeScript II (TaKaRa Inc., Dalian, China) reverse transcription kit and random hexamer primers (TaKaRa). The editing efficiency of RNA was primarily measured by sequencing the RT-PCR product according to previously reported procedures [48,49]. The level of RNA editing at each site was measured by the relative height of the nucleotide peaks in the sequencing results. A statistical analysis was performed using four replicates.

### 4.12. Subcellular Localization Experiment

For a subcellular localization, the cDNA sequence of *SPL42* was amplified from the wild type and introduced into the vector pCAMBIA1305.1-GFP in frame with the N terminus of GFP. The recombinant vector was transformed into Agrobacterium EHA105 and subsequently transformed into the 5- to 6-week-old tobacco leaves by an A. tumefaciens-mediated method as described previously [50]. Similarly, the full-length cDNA as cloned into the transient expression vector pAN580 with the CaMV35S promoter. The recombinant vector was used to transform rice protoplasts as described previously [51]. GFP fluorescence signals were observed with a confocal laser scanning microscope (LSM780, Carl Zeiss, Germany) [50]. The primers for a subcellular localization were listed in Table A4.

### 4.13. Bimolecular Fluorescence Complementation Assay

The full-length cDNA fragments of *SPL42* and *OsMORF8-1* and *OsMORF8-2* and *OsTP85* were cloned into the linearized pSPYNE173 and Pspyce (M) vectors, respectively. The primers used are listed in Table A4. Different combinations of Agrobacterium containing the above plasmids were co-transformed into *Nicotiana benthamiana* leaves [50]. After 48 h, YFP fluorescence was observed in the leaves using an LSM780 (Carl Zeiss, Oberkoche, Germany) confocal laser scanning microscope.

### 4.14. Yeast Two-Hybrid Assay

The full-length coding region of *SPL42* was cloned into the pGBKT7 vector as prey. Then, the full-length coding region of *OsMORF8-1*, *OsMORF8-2,* and *OsTP85* were cloned into the pGADT7 vector as the bait plasmid vectors. For the interaction test, each bait construct was co-transformed with each prey construct into the yeast strain AH109, plated on SD/-Trp-Leu medium, and grown at 30 °C for 2 days. Then, co-transformed yeast clones were serially diluted (1:10, 1:100, 1:1000) and spotted and grown on SD/-Leu/-Trp/-His/-Ade medium at 30 °C for 4 days. Empty vectors were co-transformed as the negative controls. The primers used are listed in Table A4.

### 4.15. RNA-Sequencing Analysis

mRNA was purified from the total RNA that was extracted from the third leaves of the wild-type and the *spl42* seedlings and sent to Beijing Biomarker Biotechnology Co. Ltd. (http://www.biomarker.com.cn/, accessed on 15 December 2022) for sequencing and analysis. The criteria for significant differentially expressed gene screening were: |log^2^Ratio| ≥ 1 and q-value ≤ 0.05. The gene ontology (GO) functional analysis was performed on the Blast2GO program [52]. A pathway enrichment analysis was conducted using the Kyoto Encyclopedia of Genes and Genomes database [53]. All requirements for the RNA-sequencing reads will be provided in a timely manner by the corresponding author.

## Data Availability

Sequence of the *spl42* mutant gene has been deposited in NCBI (https://www.ncbi.nlm.nih.gov, accessed on 15 December 2022) with GenBank accession number OQ186605.

## Appendix A

**Table A1:** Agronomic traits of wild type (WT) and *spl42*.

Agronomic Traits	WT	*spl42*
Plant height (cm)	89.75 ± 1.58	85.70 ± 2.10 **
Panicle length (cm)	18.64 ± 1.54	16.92 ± 1.21 **
Effective tiller number	5.8 ± 1.33	5.75 ± 1.92
Flag leaf length (cm)	28.99 ± 2.87	24.13 ± 0.83 **
Flag leaf width (cm)	1.8 ± 0.13	1.71 ± 0.07 *
Setting rate (%)	0.92 ± 0.05	0.83 ± 0.07 **
Grain length (mm)	7.58 ± 0.37	7.57 ± 0.37
Grain width (mm)	3.1 ± 0.2	3.1 ± 0.23
Thousand grain weight (g)	26.12 ± 0.06	26.35 ± 0.24
Maximum light quantum efficiency	0.81 ± 0.01	0.80 ± 0.003
Quantum yield	626 ± 64.78	496 ± 28.28 **
Photosynthetic rate (μmol CO^2^ m^−2^ s^−1^)	9.24 ± 0.57	5.45 ± 0.77 **
Conductance to H_2_O (mol H_2_O m^−2^ s^−1^)	0.27 ± 0.04	0.18 ± 0.01 **
Transpiration rate (mmol H_2_O m^−2^ s^−1^)	5.27 ± 0.46	3.84 ± 0.07 **

For 20 replicates, analysis of difference significance is based on Student’s *t*-test, * *p* < 0.05; ** *p* < 0.01.

**Table A2:** Peroxide related index content of wild type and *spl42*.

Peroxide Related Index	Wild Type	*spl42*
H_2_O_2_ content (μmol/g)	611.67 ± 7.70	1044.60 ± 13.38 **
CAT activity (U/g)	1436.05 ± 139.42	326.61 ± 18.33 **
SOD activity (U/g)	1755.19 ± 23.39	2321.24 ± 28.36 **
POD activity (U/g)	12,306.67 ± 231.71	29,966.67 ± 124.72 **
MDA content (nmol/g)	5.04 ± 0.62	8.65 ± 1.23 **

For 3 replicates, the analysis of difference significance is based on Student’s *t*-test, ** *p* < 0.01.

**Table A3:** The putative function of fourteen different genes in 123 kb mapping region.

ORFs	Locus	Functional Description
ORF1	*Os02g0168000*	Conserved hypothetical protein
ORF2	*Os02g0168100*	Similar to 4-hydroxyphenylpyruvate dioxygenase (EC 1.13.11.27) (4HPPD) (HPD) (HPPDase)
ORF3	*Os02g0168200*	Similar to transfactor-like protein
ORF4	*Os02g0168300*	Hypothetical protein
ORF5	*Os02g0168400*	Hypothetical protein
ORF6	*Os02g0168500*	Conserved hypothetical protein
ORF7	*Os02g0168600*	Ovarian tumor, otubain domain containing protein
ORF8	*Os02g0168700*	Peptidylprolyl isomerase, FKBP-type domain containing protein
ORF9	*Os02g0168800*	Similar to Porphobilinogen deaminase (Fragment)
ORF10	*Os02g0168900*	Conserved hypothetical protein
ORF11	*Os02g0169000*	Conserved hypothetical protein
ORF12	*Os02g0169300*	Similar to Phosphoglycerate kinase, cytosolic (EC 2.7.2.3)
ORF13	*Os02g0169400*	Similar to Argonaute 4 protein
ORF14	*Os02g0169700*	Glycine cleavage H-protein family protein

**Table A4:** List of primer sequences (5′→3′) used in this study.

Primers for map-based cloning	
RM6367-F	CAGACAGAACAGCGGTCAAG
RM6367-R	GATGGATGGATGGATTGGAG
RM5622-F	CGAAACAAGCAGCTCTAGGG
RM5622-R	CATGTCGAATTGTGGTGAGG
2-2.9-3-F	AGGGCATGAAAGCAGCTCAA
2-2.9-3-R	TGATCGTTCCCAAGCATCCC
2-3.3-2-F	AGAACCCGGTCCTTCTGAAC
2-3.3-2-R	AAGGGAAGTCTCTGCTTGCC
2-3.6-1-F	CTGCTAGCCTCACACCTACG
2-3.6-1-R	CATCTGATGCATGCACGTCG
2-3.6-3-F	AGCCAAAGTTACAGTAGCATGGA
2-3.6-3-R	GATTGGCGCCACCAGAAAAT
2-3.7-1-F	TTGCAGGTAGCCACAGGATT
2-3.7-1-R	GTTCGTGCAGCTTGAGACCA
2-3.7-3-F	AAATCTGAGTTTATTGGGGGAGTTT
2-3.7-3-R	CAGCAACCTGGAGGCTAAAAG
2-3.8-3-F	TGTGTGTTGACGATTGCTGAA
2-3.8-3-R	AAGGCCTAAGGGGCGAAAAA
Primers for transgenic constructs	
HB-F	CCGGCGCGCCAAGCTTATCTCACTGCCAAGTACTCCT
HB-R	GAATTCCCGGGGATCCGACAGCTCAACATTGTGGTACA
Crispr-F	GGCACCTTGCCACCTATATCTGCC
Crispr-R	AAACGGCAGATATAGGTGGCAAGG
Primers for yeast two hybrid	
*SPL42*-*BD*-F	CATGGAGGCCGAATTCATGCCGCCGCCGCCGAGATG
*SPL42*-*BD*-R	GCAGGTCGACGGATCCTCATTGCAAGCTATCAAAGA
*MORF8-1*-*AD*-F	GGAGGCCAGTGAATTCATGGTGTCGGCGTCGC
*MORF8-1*-*AD*-R	CGAGCTCGATGGATCCCTACTGGTAATTCCTCCCTGGCT
*MORF8-2*-*AD*-F	GGAGGCCAGTGAATTCATGGCGTCGGCGTCG
*MORF8-2*-*AD*-R	CGAGCTCGATGGATCCCTACTGGTAATTCCTCCCTTGTCCG
*OsOTP85*-*AD*-F	GGAGGCCAGTGAATTCATGTCGTCCTCGCCTCTAACC
*OsOTP85*-*AD*-R	CGAGCTCGATGGATCCCTACCAGTAGTCACCACAAGAGCA
Primers for bimolecular fluorescence complementation (BiFC)	
*YC*-*SPL42*-F	CATTTACGAACGATAGTTAATTAAATGCCGCCGCCGCCGAGATG
*YC*-*SPL42*-R	CACTGCCACCTCCTCCACTAGTTTGCAAGCTATCAAAGAAGC
*YN*-*MORF8-1*-F	CATTTACGAACGATAGTTAATTAAATGGTGTCGGCGTCGC
*YN*-*MORF8-1*-R	CACTGCCACCTCCTCCACTAGTCTGGTAATTCCTCCCTGGCT
*YN*-*MORF8-2*-F	CATTTACGAACGATAGTTAATTAAATGGCGTCGGCGTCG
*YN*-*MORF8-2*-R	CACTGCCACCTCCTCCACTAGTCTGGTAATTCCTCCCTTGTCCG
*YN*-*OsOTP85*-F	CATTTACGAACGATAGTTAATTAAATGTCGTCCTCGCCTCTAACC
*YN*-*OsOTP85*-R	CACTGCCACCTCCTCCACTAGTCCAGTAGTCACCACAAGAGCA
Primers for subcellular localization	
*PAN580*-*SPL42*-F	CGGAGCTAGCTCTAGAATGCCGCCGCCGCCGAG
*PAN580*-*SPL42*-R	TGCTCACCATGGATCCTTGCAAGCTATCAAAGAAGCCAGGG
*TR2M*-*SPL42*-F	CACCAAATCGTCTAGAATGCCGCCGCCGCCGAGATG
*TR2M*-*SPL42*-R	TCGAGACGTCTCTAGATTGCAAGCTATCAAAGAAGC
Primers for quantitative real-time RT-PCR	
*SPL42*-F	TGCTTCTCGTGACAAGGATGGG
*SPL42*-R	ACTGTAGATCCATCAGGTGAAGCC
*UBQ5*-F	ACCACTTCGACCGCCACTACT
*UBQ5*-R	ACGCCTAAGCCTGCTGGTT
*HEMA*-F	GATGCAATCACTGCTGGAAAGCGT
*HEMA*-R	CCATCTTGCCAGCACCAATCAACA
*HEML*-F	AGAACAAAGGGCAGATTGCTGCTG
*HEML*-R	TGTTTCGTCAAGTCACGGAGAGCA
*HEMB*-F	TGGCATTGTCAGGGAAGATGGAGT
*HEMB*-R	CCAAAGCAGCACGTATTGCTCCAA
*HEMC*-F	TCATTCCGAGGGCTATTGGCTTCA
*HEMC*-R	ACACTCTAGTTGGCCAATGGTGGA
U*RO-D*-F	AGGCTTCCACTGACAGGTGTTGAT
U*RO-D*-R	AAAGAACGCCAGGGTCAACATTCC
*HEME*-F	AATGGAGGCTTGCTTGAGCGAATG
*HEME*-R	TTGTTACCAAGGCGTCTCCTTCCA
*HEMF*-F	ACTGACTGCACGATGGCAGTATGA
*HEMF*-R	AGAGATCGAGCCATTCCTTTGGGT
*CHLD*-F	TAGCACAGCTGTCAGAGTGGGTTT
*CHLD*-R	TTGCCAGCCACCTCAAGTATCTCA
*CHLH-*F	GCACGGGAACTTGGCGTTTCATTA
*CHLH*-R	ACATGTCCTGGAGCTGCTTCTCAT
*CHLI*-F	AGGGATGCTGAACTCAGGGTGAAA
*CHLI*-R	AAGTAGGACTCACGGAACGCCTTT
*CHLM*-F	GCTTCATCTCCACGCAGTTCTACT
*CHLM*-R	GCAATGACGAATCGAAGACGCACA
*CRD*-F	TGGATCTAACATGACACGCACCCA
*CRD*-R	ACTGTAACGGCATTCTTCTCCGGT
*DVR*-F	TTCTTCGAGAGGGTGATCAGGGAA
*DVR*-R	GAAACTGGCAATGGCAGCCAAGAA
*POR*-F	TCGTCGGCCTCGTCTGAGTTTATT
*POR*-R	AGGCCTCTCTCACTGAAAGCTGAA
*CHLG*-F	CCAGCCACTGATGAAAGCAGCAAT
*CHLG*-R	AGAGCGCTAATACACTCGCGAACA
*psaA1*-F	GGGAGGTGGCGAGTTAGTAG
*psaA1*-R	AATGCGTGAATGTGATGGAC
*PsaA2*-F	TTATCTTCAACGAGCGGT
*PsaA2*-R	TATCTCCAGGTCCTATTGTT
*psbD1*-F	CTGCTACTGCTGTTTTCT
*psbD1*-R	GATGTTATGCTCTGCCTG
*ndh2*-F	ATCACTGTAGGACTTGGGTT
*ndh2*-R	TTTCCAGAAGAAGATGCC
*ndh4*-F	TCCTTATTGCTTATGCTGTC
*ndh4*-R	CCGTATGCTCCCATCTTTA
*RCA*-F	AAGATGACGATCGAAAAGC
*RCA*-R	AGTACTGCTCAGCCAGCTG
*RbcL*-F	AATGCGACTGCAGGTACA
*RbcL*-R	GTCATGCATTACAATAGGAA
*atpA*-F	TCAAAAAGGGCAAGATGT
*atpA*-R	TTGTAATGTAGCAGGGGAAT
*atpB*-F	ATGAATGTTATTGGTGAGCCA
*atpB*-R	CTGTTCTGTGGCTTGCTCAA
*rpoB*-F	GTGGGGAACTTGCTTTAGG
*rpoB*-R	GCTTGTTGTATCCGTCTGA
*rpoC1*-F	CATAGATTAGGCATACAGGC
*rpoC1*-R	AATAGCGGGAGATAGGAG
*APX1*-F	AGGTGCCACAAGGAAAGATCTGGT
*APX1*-R	TCAGCAGGGCTTTGTCACTAGGAA
*APX2*-F	TCAGCAGGGCTTTGTCACTAGGAA
*APX2*-R	TCCGCAGCATATTTCTCCACCAGT
*SOD-A*-F	ATCTGGATGGGTGTGGCTAGCTTT
*SOD-A*-R	AGTACGCATGCTCCCAGACATCAA
*CATA*-F	CAACCGCAACGTCGACAACTTCTT
*CATA*-R	TTCACCGGCAGCATCAGGTAGTTT
*CATB*-F	GCTTGCTTTCTGCCCAGCGATAAT
*CATB*-R	AAATAGTTTGGGCCAAGACGGTGC
*AOX1a*-F	CTTCGCATCGGACATCCATTA
*AOX1a*-R	TCCTCGGCAGTAGACAAACATC
*AOX1b*-F	CCTGCTCAGTTCATCACCATCA
*AOX1b*-R	GCATAAAACGGAGTGACAATAGC
*AOX1c*-F	GCGTCGGATGTTCATTTTCA
*AOX1c*-R	CCGCCGTAAGATTTCTTCAGT
*Osl2*-F	GCAGACAACAAATCGCCAAAT
*Osl2*-R	TCTCCAGCAACTCTAACCAGCAT
*Osl30*-F	GAGAAATCCCTTGAAGCCAA
*Osl30*-R	CACAAAGCAGTGAAAGCACA
*Osl43*-F	ATTCAGCATTCCACTGCAAG
*Osl43*-R	ATTCAGCATTCCACTGCAAG
*Osh36*-F	GTGCACCATGCACTTAATCC
*Osh36*-R	CACCGACCCTTCCTGTAGTT
*Osh69*-F	ACGAGCTACACGCCTACCTT
*Osh69*-R	ACTTCCTTGCCAGAAGCACT
*OsMC1*-F	GCTTCATCAAGGCGGTGGAGTG
*OsMC1*-R	AAGTTGGCGACCTTGCGGATG
*OsMC2*-F	CGACCCGTACAGGGTGCCGA
*OsMC2*-R	GCACAGCGCCTCGTCGTAGC
*OsMC3*-F	GGCTCCTTCGTCCGCAAGAT
*OsMC3*-R	CACAGGAGAAACGGTTTCCTGT
*OsMC4*-F	TCGACGTTCGTGGAGATGCTC
*OsMC4*-R	ATTCACGAGCCGCCTGATCTT
*OsMC5*-F	GTGCCAGACCGACCAGACAT
*OsMC5*-R	CCGCTCTTCTCCGACAGGAT
*OsMC6*-F	CCACACCGCAGGGTTCTTCAT
*OsMC6*-R	GTCCAGGCTGCTGAGTGTATCC
*OsMC7*-F	ATACAGACCGTGCTGGCGTC
*OsMC7*-R	AGGAATGGCGTCTCGGCGTT
*OsMC8*-F	TCCGGCAAGTGCCTCGTAAC
*OsMC8*-R	CAATGCGGTCGGTCACAGGAT
Realtime PCR primers to verification RNA-seq	
*Os05g0474800*-F	GACGTCCAACTCCTTCATGC
*Os05g0474800*-R	CATGGTCGGTTCTTGGTAGC
*Os04g0103500*-F	CTCCATGCTCTGCTGCAAAT
*Os04g0103500*-R	ACTTGCCGTTGTTCGAGATG
*Os07g0130400*-F	GGATACCTAGCGCCAGAACT
*Os07g0130400*-R	TCTTCGCCCACAAGTAACCT
*Os06g0545900*-F	AAGGCTTCTGCAAATGGCAA
*Os06g0545900*-R	CCATTCGCGCTGATCAGAAT
*Os07g0283125*-F	TGCTCCTGCAGCTCCTATAC
*Os07g0283125*-R	TGGTCAGGTTAGCACCAGAG
*Os05g0112800*-F	CGTCTGGTACGACTACTGCT
*Os05g0112800*-R	TTCTGCGTGTTGAGGAGGAT
*Os07g0620400*-F	CTGGCTAGGCATCTTCACCT
*Os07g0620400*-R	CGGTGCGTTTACTCCTTTCC
*Os02g0100100*-F	GCGTTCCCAAGGACTACAAC
*Os02g0100100*-R	CGAAGCAGCAGCAATCTCAT
*Os09g0416200*-F	GGTGTTCCGGAAGAAGAACG
*Os09g0416200*-R	GAGGTAGAGGGAGGAGGTGA
*Os04g0581000*-F	AGCTGTATTCCGATGAGCCA
*Os04g0581000*-R	TCGGCAGTACGTGCTCATAA
*Os04g0577800*-F	GGCTCCATCTTTGGAGGACT
*Os04g0577800*-R	TCCAAATCCAACAGCGTCAC
*Os09g0517000*-F	GCCAGAGGAGAGGAAGGAC
*Os09g0517000*-R	CTCATCTTGGCCACGAACTC
*Os03g0304500*-F	TCCAAGAATCCCAACACGGA
*Os03g0304500*-R	GAAGAGAAGCTGTCCTCCGA
*Os07g0176900*-F	GTTGTGCAGTTCTGCTGGAA
*Os07g0176900*-R	GTCGGTAACGTAGGGTTTGC
*Os07g0630800*-F	ATCTTGGACACTGCAACACG
*Os07g0630800*-R	GTTGATGCCGAACAGGTCAT
*Os07g0634600*-F	CCAATGCCAACTCTGGTAGC
*Os07g0634600*-R	CCTCGGTGTAGGTGATACCC
*Os03g0366350*-F	AAGGCCAACAACAACAGCAA
*Os03g0366350*-R	TGACGACGTTCTTCCTCTCC
*Os12g0115300*-F	GATGGTGATGGCGATGGTG
*Os12g0115300*-R	CCGTCACGTAAGAGATGCAC
*Os10g0533500*-R	ATCGAGAACGTGCCCTACTT
*Os10g0533500*-F	CATGGCCTCCTTCCTCTTGA
*Os05g0374200*-F	GCTCCTGTTACTTGCCCTTG
*Os05g0374200*-R	ATCGTGGAAGCTGAAATGGC
Primers for RNA editing site in chloroplast	
*atpA*-F	CCCAGGGGATGTTTTTTATT
*atpA*-R	TGAAAAAAGCGTCCATTGTC
*ndhD*-F	ATTTTGGCTTCCTTATTGC
*ndhD*-R	GCCTCTACCCTGTCAACG
*ndhF*-F	ATATGCATGGGTAATCCCTC
*ndhF*-R	AGTGGCTCCTAAGAAAAGTG
*ropB*-F	ACTAAGCGTGCTATTCTCAA
*ropB*-R	TTTATGGTCTAATTCCGAGC
*rps14*-F	ATGGCAAAAAAAAGTTTGATTC
*rps14*-R	TTACCAACTGGATCTTGTTGCA
*rpoC2*-F	GGTCCTTGGGGATTCTTGAT
*rpoC2*-R	TCTTGTTTTGTGGGTAACGG
